# UBAP2L arginine methylation by PRMT1 modulates stress granule assembly

**DOI:** 10.1038/s41418-019-0350-5

**Published:** 2019-05-21

**Authors:** Chuyu Huang, Yan Chen, Huaiqian Dai, Huan Zhang, Minyu Xie, Hanbin Zhang, Feilong Chen, Xiangjin Kang, Xiaochun Bai, Zhenguo Chen

**Affiliations:** 1 Department of Cell Biology, School of Basic Medical Sciences, Southern Medical University, Guangzhou, 510515 P. R. China; 2 Center for Reproductive Medicine, Department of Obstetrics and Gynecology, Nanfang Hospital, Southern Medical University, Guangzhou, 510515 P. R. China; 3 Center for Reproductive Medicine, Third Affiliated Hospital of Guangzhou Medical University, Guangzhou, 510515 P. R. China

**Keywords:** RNA, Cell biology

## Abstract

Stress granules (SGs) are discrete assemblies of stalled messenger ribonucleoprotein complexes (mRNPs) that form when eukaryotic cells encounter environmental stress. RNA-binding proteins (RBPs) mediate their condensation by recruiting populations of mRNPs. However, the cellular and molecular mechanisms underlying the role of ubiquitin-associated protein 2-like (UBAP2L) in the regulation of SG dynamics remain elusive. Here, we show that UBAP2L is required for both SG assembly and disassembly. UBAP2L overexpression nucleated SGs under stress-null conditions. The UBAP2L Arg–Gly–Gly (RGG) motif was required for SG competence, and mediated the recruitment of SG components, including mRNPs, RBPs, and ribosomal subunits. The domain of unknown function (DUF) of UBAP2L-mediated interaction with ras GTPase-activating protein-binding protein (G3BP)1/2, and its deletion caused the cytoplasmic–nuclear transport of UBAP2L and G3BP1/2, thereby compromising SG formation. The protein arginine methyltransferase PRMT1 asymmetrically dimethylated UBAP2L by targeting the RGG motif. Increased arginine methylation blocked, whereas its decrease enhanced UBAP2L interactions with SG components, ablating and promoting SG assembly, respectively. These results provide new insights into the mechanisms by which UBAP2L regulates SG dynamics and RNA metabolism.

## Introduction

Stress granules (SGs) are cytoplasmic foci that are assembled when untranslated messenger ribonucleoprotein complexes (mRNPs) accumulate in eukaryotic cells exposed to biotic stress (e.g., viral infections) or environmental stress (e.g., oxidation, heat, or starvation) [1]. SG formation typically follows translational inhibition, due to canonical stress-induced phosphorylation of eukaryotic initiation factor (eIF)2α, or noncanonical disaggregation of the cap-binding eIF4F complex (consisting of eIF4E, eIF4A, and eIF4G) (p-eIF2α independent) [2, 3]. Therefore, SGs are commonly composed of mRNAs, small ribosomal subunits, eIFs, and RNA-binding proteins (RBPs).

SG assembly is a complex process involving multiple RNA–RNA, RNA–protein, and protein–protein interactions [4]. Two models have been proposed to explain the multiple phases of SG assembly, namely the “Cores First” model and the “Liquid–Liquid Phase Separation (LLPS) First” model [4–6]. SGs are also dynamic structures. First, they exhibit liquid-like behavior and rapid exchange rates of components. Second, they are disassembled into translating mRNPs upon recovery from stress. Third, various post-translational modifications (PTMs) in SG-nucleating proteins modulate SG dynamics, including phosphorylation [7, 8], dephosphorylation [9], poly(ADP)ribosylation [10], deacetylation [11], and glycosylation [12]. Recent evidence suggests that methylation is an important PTM that regulates SG dynamics [13–15]. Protein arginine methyltransferases (PRMTs) methylate arginine residues in glycine and arginine-rich motifs. To date, nine mammalian PRMTs have been identified, and are divided into three types [16, 17]. Of those, PRMT1, which belongs to type I PRMTs that catalyze the formation of asymmetric dimethylarginine (ADMA), regulates SG formation by methylating multiple SG-nucleating proteins, including FUS/LTS [18, 19], G3BP1 [20], and G3BP2 [21], and thus may play a major role. However, the molecular networks governing SG assembly and disassembly remain unclear.

Ubiquitin-associated protein 2-like (UBAP2L) is a highly conserved protein with an N-terminal ubiquitin-associated (UBA) domain involved in the ubiquitin–proteasome system and aggregate formation induced by proteasome inhibitors [22]. It was originally identified as a human sperm protein that interacts with zona pellucida 3 in human eggs [23]. It can interact with BIM1 to form a complex that modulates hematopoietic stem cell activity [24]. Numerous studies indicated its association with various types of cancer [25–30]. It is required for the accurate distribution of chromosomes during mitosis [31]. A recent study suggested that it is involved in SG formation [32]; however, the cellular and molecular mechanisms underlying the role of UBAP2L in the regulation of SG assembly and disassembly need to be further investigated. In this study, UBAP2L recruited SG components through its Arg–Gly–Gly (RGG) motif, which is essential for SG competence. The RGG motif in UBAP2L was asymmetrically dimethylated by the arginine methyltransferase PRMT1, and increased arginine methylation of UBAP2L inhibited SG assembly. We propose a model to explain the function of UBAP2L in SG condensation.

## Materials and methods

### Plasmids

All plasmids were constructed by Genechem Co., Ltd (Shanghai, China). A cDNA clone was purchased from GeneCopoeia (cDNA clone MGC: 4404, IMAGE: 2906083) for full-length human UBAP2L (NCBI accession no. BC003170.1). The coding region of UBAP2L was cloned into the pcDNA3.1 vector, in-frame with a Flag tag within the vector. This plasmid (wild type) was preserved as the seed for the generation of Flag-tagged UBAP2L mutants (Supplementary Fig. S1), including the following deletants: UBAP2L with deleted amino acids 2–130 (Δ2–130, lacking the UBA domain), Δ131–190 (lacking the RGG motif), Δ239–290 (lacking the two putative RNA-binding regions), and Δ495–526 (lacking the DUF3695 domain); and the following point mutants: UBAP2L with all Arg residues in the RGG domain mutated to Lys (UBAP2L-R_131–190_K), all Arg mutated to Ala (UBAP2L-R_131–190_A), only the Arg-187, or Arg-190, or both mutated to Ala (R_187_A, R_190_A, and R_187+190_A, respectively). The cDNA for human G3BP2 (NM_203504.2) and for human PRMT1 (NM_001536) was chemically synthesized and cloned into the pcDNA3.1 vector with a HA tag.

### Cell culture and transfections

HEK293, HeLa, and PC3 cells were maintained in a 5% CO_2_, 37 °C humidified incubator, and cultured in DMEM supplemented with 10% FBS (AusGeneX, Brisbane, Australia). Where stated, plasmids (48 h) or pooled siRNAs (72 h, sequences listed in Supplementary Table S1; GenePharma, Shanghai, China) were transfected using Lipofectamine 3000 (Invitrogen, Carlsbad, CA, USA). Cells were then treated with/without the indicated SG-inducing chemicals, and finally subjected to western blotting (WB), immunofluorescence, and immunoprecipitation assays. In some experiments, HeLa cells were infected with lentiviral shRNA (Genechem) targeting human *UBAP2L*, and selected with 5 μg/ml puromycin at 72 h after infection. Five days post infection, shRNA-expressing cells were subjected to subsequent treatments and analyses.

### Chemical reagents, SG induction, and quantification

Chemical reagents used for SG induction included sodium arsenite (AS), clotrimazole (CZ), H_2_O_2_, sorbitol (all from Sigma-Aldrich, Shanghai, China), and NaCl (Guangzhou Chemicalreagent Co., Ltd, Guangzhou, China). SGs were induced by treatment with AS (500 μM for 1 h, unless specifically indicated in the figure legends), CZ (20 μM for 1 h), H_2_O_2_ (1 mM for 1 h), NaCl (0.2 M for 30 min), or sorbitol (0.4 M for 30 min), or recovered from stress for 1 h. Cells were scored for SGs by manual counting using fluorescence microscopy with G3BP and FXR1 or eIF4G as SG markers; only cells with granules co-stained for these markers were considered SGs, and a minimum of three granules per cell was required for a positive score. In the experiments with Flag-tagged UBAP2L deletants or mutants, only Flag-positive cells were considered, and the number of granules in an individual cell was counted. At least 100 cells from at least five fields were analyzed.

### Immunofluorescence (IF)

Cells were grown on glass-bottom cell culture dishes (Nest), stressed as indicated, and fixed using 4% paraformaldehyde in PBS for 15 min, followed by 5 min of permeabilization in 0.1% Triton X-100/PBS. Cells were blocked in 5% normal goat serum/PBS for 20 min, and then incubated with primary antibodies overnight at 4 °C. Alexa Fluor 488- or 594-labeled secondary antibodies (Jackson Immunoresearch, West Grove, PA, USA) were used for secondary incubations, and 4,6-diamidino-2-phenylindole (DAPI) was used to visualize the nuclei. Immunofluorescent images were obtained using a FluoView FV1000 confocal microscope (Olympus, Tokyo, Japan). The primary antibodies used for IF analyses are summarized in Supplementary Table S2.

### Western blotting

Protein lysates were subjected to 6–12% SDS-PAGE and electrotransferred to nitrocellulose membranes (GE Healthcare Life Sciences, Beijing, China). The membranes were then blocked in 5% nonfat dry milk for 1 h at room temperature, washed, and incubated with the indicated primary antibodies overnight at 4 °C. The membranes were further washed, incubated with horseradish peroxidase (HRP)-conjugated secondary antibodies (Jackson Immunoresearch) for 1 h at room temperature, washed again, and finally visualized using an enhanced chemiluminescence kit (PerkinElmer, Waltham, MA, USA). The primary antibodies used for WB analysis are summarized in Supplementary Table S2.

### Co-immunoprecipitation (Co-IP)

Immunoprecipitation assays were performed as described previously with minor modifications [33]. Briefly, confluent HEK293 cells in a 60-mm dish were treated as indicated, rinsed with cold PBS, and then lysed in cold CHAPS-containing lysis buffer [0.3% CHAPS, 40 mM HEPES (pH 7.4), 150 mM NaCl, 2 mM ethylenediamindium pyrophosphate, 10 mM sodium glycerophosphate, 50 mM NaF, and one tablet of EDTA-free protease inhibitors per 25 ml]. In some experiments, 40 μg/ml RNase A with/without EE (2 mM EDTA, 2.5 mM EGTA), and/or 5 mM MgCl_2_ were added into the lysis buffer. Cells were rotated for 20 min at 4 °C, cleared by centrifugation (12,000 rpm at 4 °C for 10 min), and incubated with the indicated primary antibodies for 2 h with continuous rotation at 4 °C. A 50% slurry of protein G Sepharose (60 μl) was then added, and the incubation continued for an additional 1 h. Immunoprecipitated proteins were denatured by addition of 50 μl of SDS loading buffer and boiling for 5 min, resolved by 6–12% SDS-PAGE, and finally analyzed by immunoblotting.

### In vitro methylation assay

In vitro methylation was conducted following combined instructions as described previously [20, 31]. Briefly, HA-PRMT1 was purified from HEK293 cells using a protein G Sepharose column, and then reacted with 1 μl of recombinant UBAP2L (Origene, Beijing, China), 80 μM S-adenosylmethionine (NEB, Beijing, China), and 1 × PBS in a reaction volume of 30 μl. The mixtures were gently agitated at 30 °C for 90 min and then subjected to SDS-PAGE and immunoblot analysis with an anti-ADMA antibody.

### Cytoplasmic and nuclear protein extraction

Cytoplasmic and nuclear protein extraction was performed using a commercial kit (Invent Biotechnologies, Beijing, China). Briefly, confluent HeLa cells plated in a 60-mm dish were treated as indicated, rinsed with cold PBS, and then lysed in cytoplasmic extraction buffer on ice for 5 min. Cell lysates were transferred to prechilled 1.5-ml microcentrifuge tubes, which were vortexed vigorously for 15 s. After centrifugation at 15,000 rpm at 4 °C for 5 min, the supernatant was collected and stored as the cytosolic fraction. After addition of appropriate amounts of nuclear extraction buffer to the pellet, the solution was vortexed vigorously for 15 s and incubated on ice for 1 min. Vortexing for 15 s and incubation for 1 min was repeated four times. After centrifugation at 15,000 rpm for 30 s, nuclear extracts were collected for further analysis.

### High-performance liquid chromatography–mass spectrometry (HPLC–MS)

Immunoprecipitated proteins were subjected to trypsin digestion, HPLC–MS/MS, and data analysis by Fitgene Biotechnology Co., Ltd (Guangzhou, China) as detailed in Supplementary Methods.

### Statistics and reproducibility

All experiments were performed in triplicate. Data are expressed as the mean ± SD. The differences in the rate of SG-positive cells were analyzed using the chi-square test. The average number of granules in a cell was compared using the *t-*test for between-group differences and the ANOVA test for more than two groups after tests of normality and homogeneity of variance, followed by the Dunnett test for between-group differences (SPSS 13.0). *P* < 0.05 was considered statistically significant.

## Results

### UBAP2L is involved in SG assembly

**Fig. 1:**
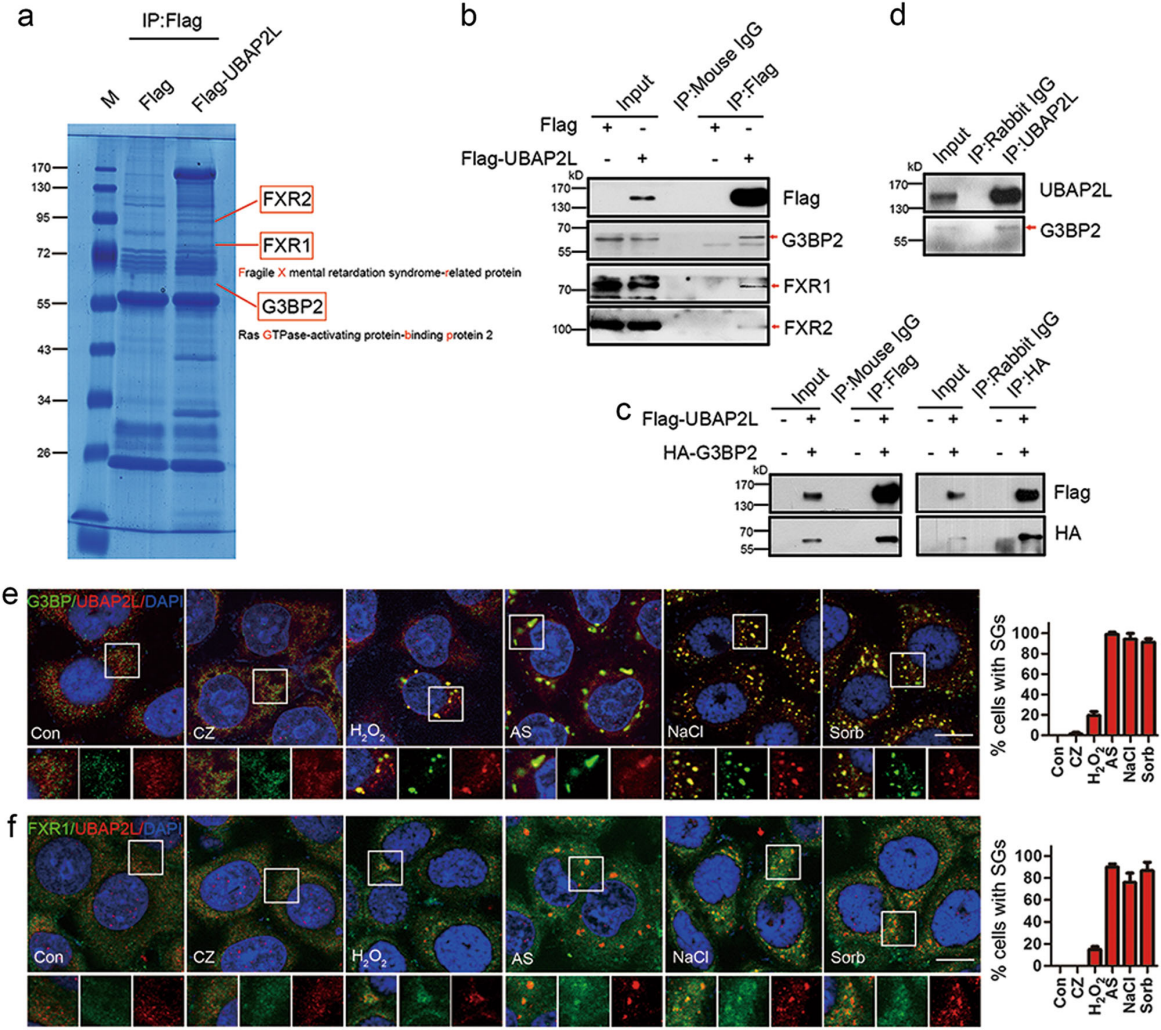
UBAP2L is involved in both p-eIF2α-dependent (AS) and p-eIF2α-independent (NaCl and sorbitol) initiation of SG formation. **a** Coomassie blue-stained gel showing three UBAP2L interacting partners identified by a Co-IP/MS assay. HEK293 cells were transfected with the Flag tag or Flag-UBAP2L plasmids. After 48 h, Co-IP was performed using a Flag primary antibody coupled with a MS assay. M, marker. **b** Confirmation of the results of (**a**) by Co-IP/WB assay. Red arrows indicate the bands corresponding to target proteins. **c** Confirmation of the interaction between UBAP2L and G3BP2 by reciprocal IP. HEK293 cells were simultaneously transfected with Flag-UBAP2L and HA-G3BP2, and reciprocal IP was performed, followed by WB analysis of HA in the Flag precipitates or of Flag in the HA precipitates. **d** Determination of the association between endogenous UBAP2L and G3BP2 by Co-IP/WB. Endogenous UBAP2L was immunoprecipitated from normal HEK293 cell extracts, followed by WB analysis of G3BP2 in the precipitates. **e** Double immunofluorescence of UBAP2L/G3BP under various stresses. HeLa cells were treated with or without 20 μM CZ, 1 mM H_2_O_2_, or 500 μM AS for 1 h, or 400 mM sorbitol or 200 mM NaCl for 30 min, and then stained for UBAP2L (red)/G3BP (green) as SG markers and scored. Blue indicates the nucleus counterstained by DAPI. Insets are shown with separated colors. Bars indicate the average percentage of cells containing more than three SGs. **f** Double immunofluorescence of UBAP2L (red)/FXR1 (green) under various stresses. The treatments were the same as those in (**e**). Data represent the mean ± SD. *n* = 3. Scale bars = 10 μm

The cellular functions of UBAP2L were examined by identifying its binding partners. For this purpose, HEK293 cells were transfected with the Flag-UBAP2L or Flag empty plasmid, and Co-IP was performed using a Flag primary antibody. Precipitates were separated by SDS-PAGE and stained with Coomassie blue, and bands that differed between the Flag-UBAP2L group and the Flag group were analyzed by mass spectrometry (MS). Three of the precipitated proteins were identified as fragile X mental retardation syndrome-related protein 2 (FXR2, ~95 kD), FXR1 (~80 kD), and ras GTPase-activating protein-binding protein 2 (G3BP2, ~60 kD) (Fig. 1a). Co-IP/WB analysis confirmed that UBAP2L precipitated endogenous FXR1, FXR2, and G3BP2 (Fig. 1b). Reciprocal experiments further confirmed the association of UBAP2L with G3BP2 (Fig. 1c). This molecular interaction was duplicated between endogenous UBAP2L and G3BP2 (Fig. 1d). These results indicated that UBAP2L interacts with FXR1, FXR2, and G3BP2.

Because FXR1, FXR2, and G3BP2 are involved in SG formation [9, 34–37], we investigated whether UBAP2L is also involved in this cellular process. HeLa cells were challenged with five different SG-inducing chemicals, and subjected to co-IF analysis of UBAP2L and its binding partner G3BP or FXR1 as SG marker to define the involvement of UBAP2L in SGs under various stress conditions. Particles double positive for UBAP2L and G3BP were not clearly observed in the control and clotrimazole (CZ) groups (Fig. 1e), indicating that UBAP2L is not involved in CZ-induced SGs. H_2_O_2_ induced the formation of double-positive SG foci at a mild level, resulting in ~20% foci-positive cells. Sodium arsenite (AS), NaCl, and sorbitol each induced >90% foci-positive cells (Fig. 1e). Similar results were obtained in Co-IF analysis of UBAP2L and FXR1 in HeLa cells (Fig. 1f) and Co-IF of UBAP2L and G3BP or FXR1 in PC3 cells (Supplementary Fig. S2). Collectively, these results indicated that UBAP2L is involved in the formation of SGs induced by various chemicals.

### UBAP2L modulates SG dynamics

**Fig. 2:**
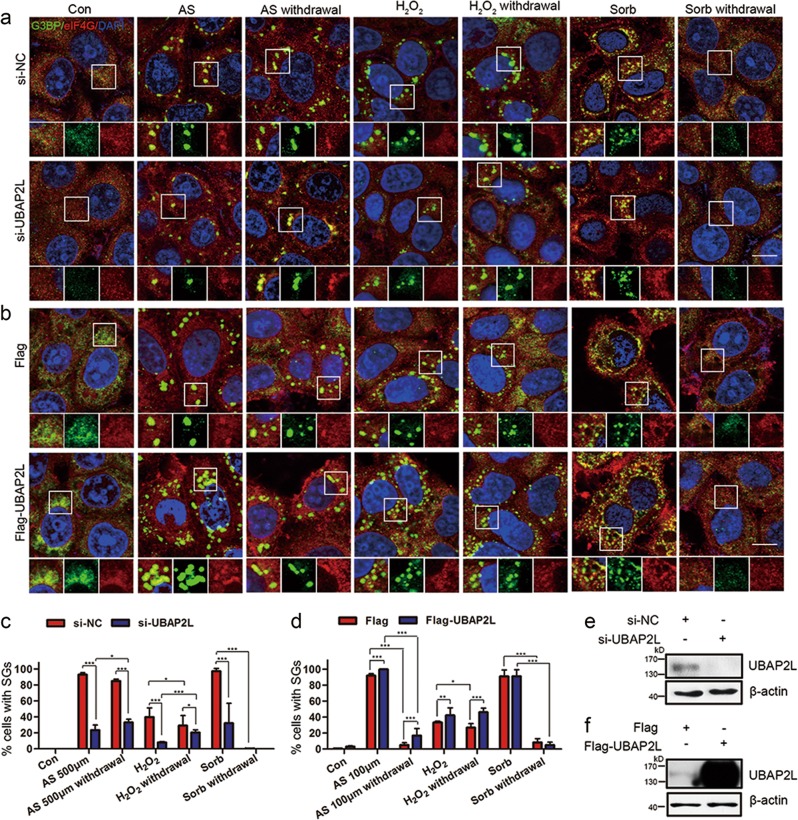
UBAP2L is required for SG assembly and disassembly. **a** SG visualization under various stresses or recovery from stresses after UBAP2L knockdown. HeLa cells were transfected with NC- or *UBAP2L*-siRNA, followed by treatment with or without AS (500 μM, 1 h), H_2_O_2_ (1 mM, 1 h), or sorbitol (400 mM, 30 min), or recovery from stress for 1 h, and then stained for G3BP (green)/eIF4G (red) as SG markers. **b** SG visualization under various stresses or recovery from stresses after UBAP2L overexpression. HeLa cells were transfected with Flag tag or Flag-UBAP2L plasmid, followed by the same treatments as in (**a**), except 100 μM AS for 1 h. **c** Quantification of SGs in (**a**). Bars indicate the average percentage of cells containing more than three SGs; between-group differences were analyzed using the chi-square test. **d** Quantification of SGs in (**b**). **e** WB analysis of UBAP2L levels in HeLa cells after UBAP2L knockdown. **f** WB analysis of UBAP2L levels in HeLa cells after UBAP2L overexpression. Data represent the mean ± SD. *P < 0.05, ***P < 0.001. *n* = 3. Scale bars = 10 μm

UBAP2L knockdown uniformly and significantly reduced the formation of AS-, H_2_O_2_−, and sorbitol-induced SGs, as assessed using G3BP and eIF4G (another typical SG component) as markers (Fig. 2a, c, and e). When sorbitol was removed, SGs in both negative control (NC) and *UBAP2L*-RNAi groups were hardly detected, indicating that sorbitol-induced SGs are dynamic. In UBAP2L knockdown cells, SG detection following AS removal was slightly higher than in those under stress. This phenomenon was more evident in the H_2_O_2_ group after recovery (Fig. 2a, c). UBAP2L knockdown by lentiviral shRNA targeting *UBAP2L* yielded similar results (Supplementary Fig. S3), suggesting that UBAP2L knockdown delayed SG degradation. SG disassembly was also monitored in HeLa cells at 1–8 h after AS withdrawal, until all SGs disassembled, which confirmed that UBAP2L knockdown delayed SG degradation (Supplementary Fig. S4). These observations indicated that UBAP2L knockdown not only inhibits stress-induced SG assembly, but also delays SG disassembly after stress removal.

UBAP2L overexpression promoted H_2_O_2_-induced SG assembly. Both the NC and RNAi group showed a >2-fold increase in SG formation after the removal of H_2_O_2_ (Fig. 2b, d, and e). However, UBAP2L overexpression did not increase sorbitol-induced SG formation or preserve SGs after H_2_O_2_ withdrawal (Fig. 2b, d), and similar results were obtained for AS at 500 μM (Supplementary Fig. S5a). The concentration of AS used (500 μM) may be too high. In cells treated with 250 μM AS, UBAP2L overexpression was only effective in retaining SGs after AS withdrawal (Supplementary Fig. S5b), whereas in response to 100 μM AS, UBAP2L overexpression effectively promoted SG assembly under stress and blocked SG disassembly after recovery (Fig. 2b, d). This may explain why UBAP2L overexpression did not increase sorbitol-induced SGs, and also indicate the heterogeneity of UBAP2L assembly under different stresses. Taken together, these results demonstrate that UBAP2L is a key modulator of SG dynamics and is required for both SG assembly and disassembly.

### UBAP2L influences the interactions between SG nucleators and 40S ribosomal subunits

**Fig. 3:**
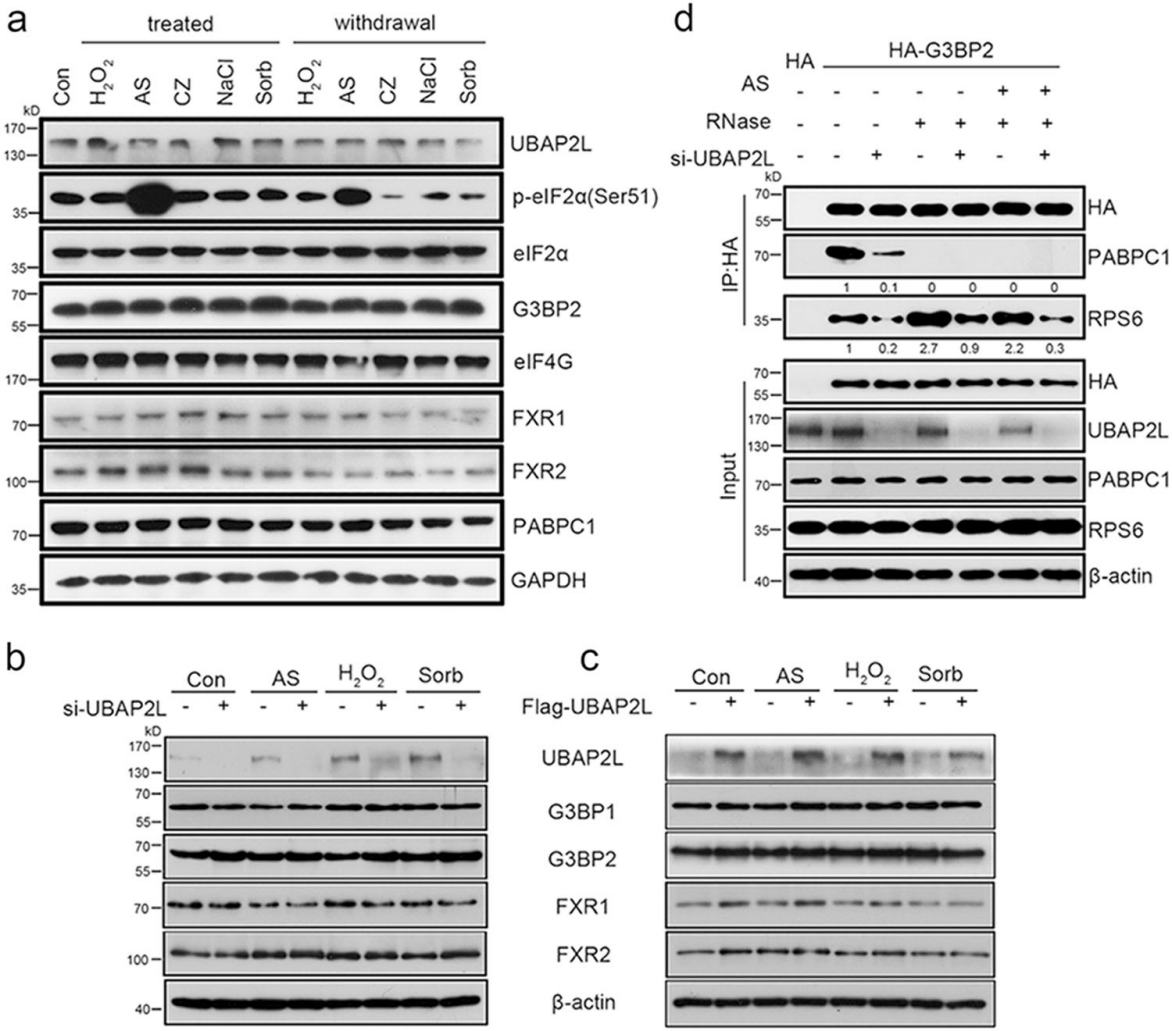
UBAP2L affects G3BP2 association with SG components. **a** UBAP2L expression under stress or recovery conditions. HeLa cells were treated with or relieved from the indicated stresses, and then subjected to WB analysis of UBAP2L, its binding partners, and other indicated SG proteins. **b** Expression of the UBAP2L binding partners in stressed HeLa cells after UBAP2L knockdown. (**c**) Expression of the UBAP2L binding partners in stressed HeLa cells after UBAP2L overexpression. **d** Co-IP/WB analysis of the associations of G3BP2 with PABPC1 and RPS6 after UBAP2L knockdown. HeLa cells were simultaneously transfected with the HA tag or HA-G3BP2 plasmid with/without *UBAP2L*-siRNA, followed by treatment with/without AS (500 μM, 1 h), in combination with/without RNase A in the lysis buffer. The HA precipitates were analyzed by WB for PABPC1 and RPS6

We next explored the mechanisms by which UBAP2L mediates SG assembly. WB analysis showed that UBAP2L protein levels remained relatively stable in the presence or absence of the stress stimulus (Fig. 3a and Supplementary Fig. S6). In addition, UBAP2L knockdown (Fig. 3b) or overexpression (Fig. 3c) did not change the protein levels of other SG proteins. This suggested that the UBAP2L-mediated regulation of SG dynamics does not involve changes in UBAP2L expression itself or that of other SG proteins. We, therefore, tested the possibility that UBAP2L affects the interactions between SG-nucleating proteins (e.g., G3BP2) and RBPs (e.g., PABPC1), or 40S ribosomal subunits [e.g., ribosomal protein (RP)S6]. As shown in Fig. 3d, UBAP2L knockdown inhibited the interaction between G3BP2 and PABPC1 or RPS6 under normal and AS conditions. Addition of RNase A abolished the association of G3BP2 with PABPC1, whereas that with RPS6 was enhanced, which was also decreased by UBAP2L knockdown. These observations demonstrated that UBAP2L affects the interactions between SG nucleators and 40S ribosomal subunits, suggesting a mechanism by which UBAP2L modulates SG dynamics.

### The RGG motif in UBAP2L is required for SG assembly

**Fig. 4:**
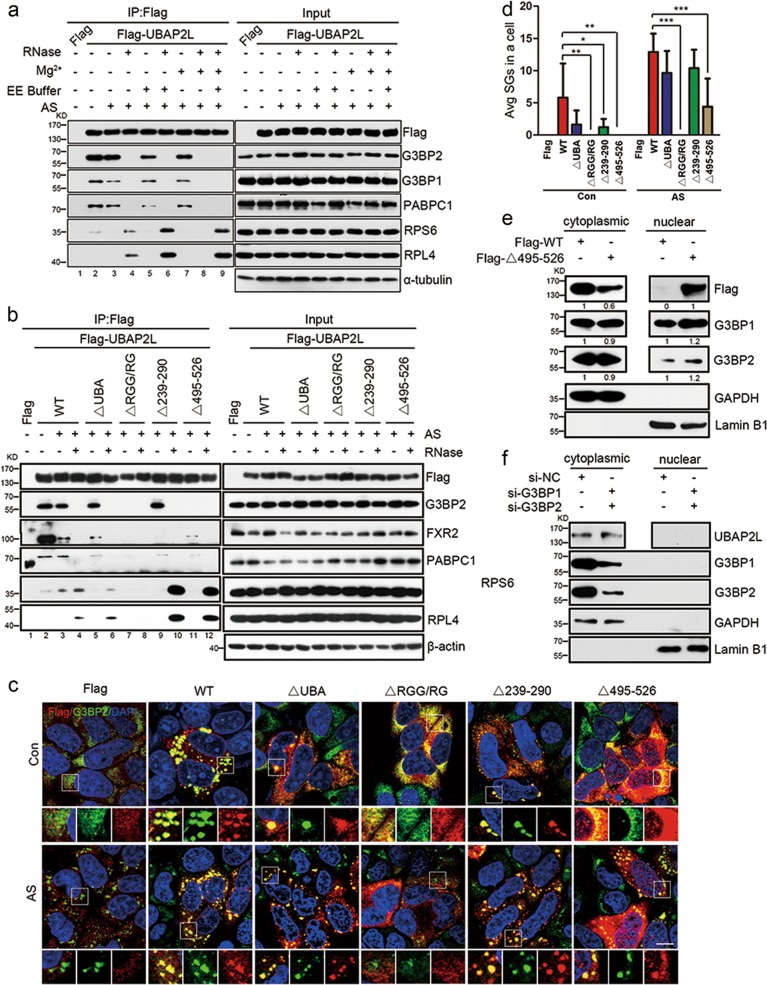
The RGG motif is required for UBAP2L SG competence. **a** Co-IP/WB analysis of the associations of UBAP2L with SG proteins. HEK293 cells expressing Flag-UBAP2L were treated with/without AS (500 μM, 1 h), in combination with/without RNase A, EE (2 mM EDTA, 2.5 mM EGTA), and/or 5 mM MgCl_2_ in the lysis buffer. The Flag precipitates were then subjected to WB analysis for the indicated SG proteins. **b** Co-IP/WB analysis of the associations of the UBAP2L deletants with SG proteins. HEK293 cells were transfected with Flag-tagged UBAP2L-wild type (WT) or deletants (depicted in Supplementary Fig. S1), followed by treatment with/without AS (500 μM, 1 h), in combination with/without RNase A in the lysis buffer. **c** SG competence in HeLa cells with a stable low UBAP2L-expressing Flag-tagged UBAP2L-WT or the indicated deletants, with/without AS (500 μM, 1 h), using Flag (red)/G3BP2 (green) as SG marker. **d** Quantification of SGs in (**c**). Bars indicate the average number of SGs in Flag-positive cells, and differences among groups were analyzed using the ANOVA test. *P < 0.05, **P < 0.01, ***P < 0.001. *n* = 3. **e** Cytoplasmic and nuclear protein fractionation assay for the expression of Flag and G3BP1/2 in UBAP2L-KD HeLa cells expressing Flag-tagged UBAP2L-WT or UBAP2L△495–526 deletant. GAPDH and Lamin B1 were used as cytoplasmic and nuclear markers, respectively. **f** Cytoplasmic and nuclear protein fractionation assay for the expression of UBAP2L in HeLa cells after double knockdown of G3BP1/2. Scale bars = 10 μm

UBAP2L contains a UBA domain (aa 49–89), an RGG motif (aa 131–190), three regions with predicted mRNA-binding activity (aa 239–257, 282–290, and 850–864), and a domain of unknown function (DUF) (aa 495–526) (Supplementary Fig. S1) [31, 32]. These features confer UBAP2L the ability to nucleate SG formation. Thus, we detected the potential associations of UBAP2L with other known SG-nucleating proteins or ribosomal subunits. Figure 4a shows that (1) UBAP2L bound G3BP1, G3BP2, and PABPC1 under normal conditions (lane 2), and these interactions decreased slightly after AS treatment (lane 3). (2) UBAP2L binding to nucleators was RNA dependent, as their associations completely disappeared upon RNase treatment (compare lanes 4, 6, 8, and 9), whereas it was resistant to ribosome-dissociating EDTA–EGTA (EE) or ribosome-stabilizing Mg^2+^ (compare lanes 3, 5, and 7). (3) UBAP2L bound to the small 40S subunit protein RPS6 under normal conditions, but not to the large 60S RPL4, which was only precipitated after RNase treatment (compare lanes 2 and 4), indicating that UBAP2L associated preferentially with the 40S subunit rather than the 60S ribosomal subunit. (4) The UBAP2L interactions with RPS6 and RPL4 were largely facilitated by RNase treatment (lane 4) and further increased by EE (lane 6), disappearing upon Mg^2+^ addition (lane 8), and rescued again by supplementation with EE to neutralize the effect of Mg^2+^ (lane 9). This indicated that UBAP2L may directly associate with RPs in EE-dissociated 40S and 60S subunits, which are not accessible in Mg^2+^-stabilized intact 80S ribosomes, but not with rRNA in subunits, a mechanism quite different from that demonstrated for G3BP [38].

To further map the domains in UBAP2L responsible for these associations, a set of truncation mutants was generated (Supplementary Fig. S1). Figure 4b shows that (1) deletion of the UBA domain or the two putative RNA-binding regions (aa 239–290) modestly decreased the association of UBAP2L with SG proteins, except G3BP2 (compare lanes 3 and 5, lanes 3 and 9). (2) Loss of the RGG motif (aa 131–190) completely abolished all tested UBAP2L interactions (compare lanes 3 and 7). (3) The DUF domain (aa 495–526) mediated UBAP2L binding to G3BP2, but not to other SG proteins (compare lanes 3 and 11), in line with previous findings in *Drosophila melanogaster* [39]. These results suggest that the RGG motif is universally required for UBAP2L-mediated SG formation by recruiting other nucleators and ribosomal subunits, and the DUF domain mediates the molecular interactions between UBAP2L and G3BP, which synergistically contribute to SG nucleation.

We next identified the role of each domain in SG condensation in HeLa cells with stable UBAP2L downregulation. The results showed that transient overexpression of UBAP2L-WT induced SG formation under normal conditions (Fig. 4c and d), suggesting that overexpression of UBAP2L nucleated SGs. UBAP2L△UBA and UBAP2L△239–290 also nucleated SGs, although they were less effective than the WT. After exposure to AS, almost all cells transfected with UBAP2L-WT were SG positive, and the cells with UBAP2L△UBA or UBAP2L△239–290 showed comparable SG competence (Fig. 4c and d). This supports the previous finding that these two regions play a modest role in SG assembly [32]. UBAP2L△RGG showed a scattered localization in the cytoplasm under normal conditions, and was completely excluded from G3BP2 assembly after AS treatment (Fig. 4c and d). This confirmed the essential role of the RGG motif in UBAP2L SG competence. Deletion of the DUF domain caused UBAP2L shuttling from the cytoplasm to the nucleus (Fig. 4c). This explained why deletion of this domain eliminated UBAP2L association with G3BP1/2, and consistently, abolished SG formation (Fig. 4d). This phenomenon was further confirmed by a cytoplasmic and nuclear protein fractionation/WB assay (Fig. 4e), which showed that deprivation of the DUF domain increased nuclear UBAP2L levels and decreased UBAP2L cytoplasmic expression. We also observed a modest cytoplasmic–nuclear translocation of G3BP1/2 (Fig. 4e). However, such UBAP2L nuclear translocation was not observed after double knockdown of G3BP1/2 (Fig. 4f). UBAP2L knockdown also failed to induce G3BP translocation into the nucleus (Fig. 2a), indicating that UBAP2L expression was not responsible for retaining G3BP in the cytoplasm, whereas the DUF domain played a role.

### The RGG motif in UBAP2L is asymmetrically dimethylated by PRMT1

**Fig. 5:**
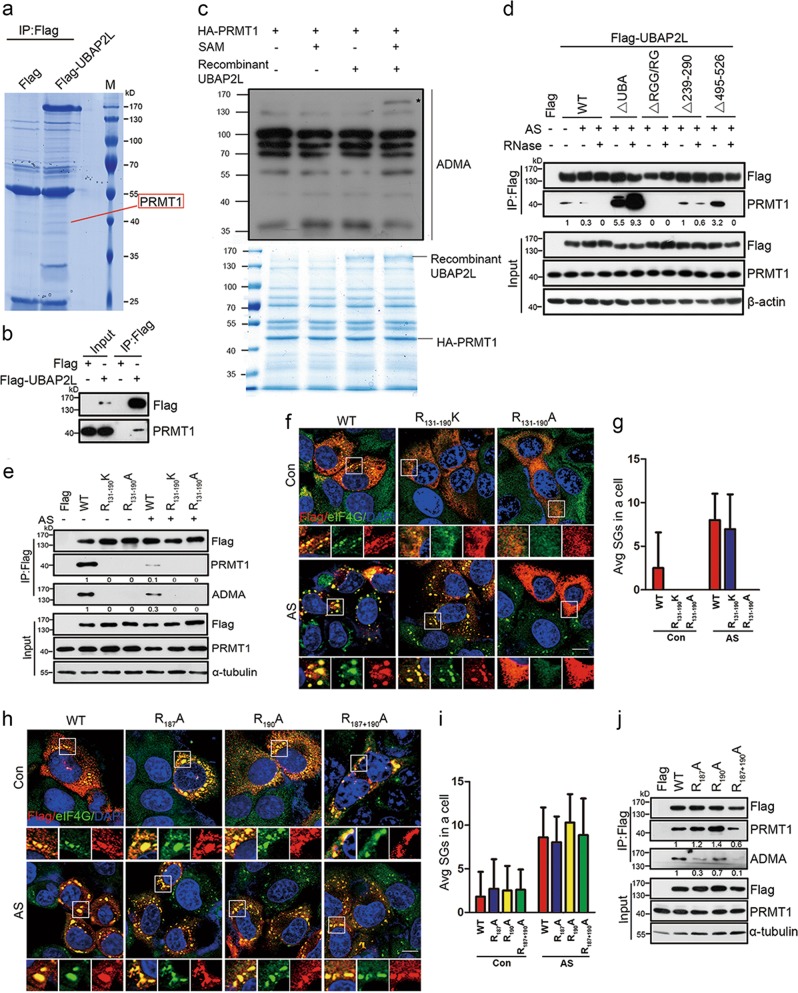
PRMT1 asymmetrically dimethylates UBAP2L by targeting the RGG motif. **a** Coomassie blue-stained gel showing that the Co-IP/MS assay identified the association of PRMT1 with UBAP2L. **b** Confirmation of the interaction between UBAP2L and PRMT1 by IP/WB. HEK293 cells were simultaneously transfected with Flag tag or Flag-UBAP2L plasmid, followed by WB analysis of endogenous PRMT1 in the Flag precipitates. **c** In vitro methylation assay on recombinant UBAP2L-mediated by PRMT1 via monitoring the ADMA level. Asterisk indicates a positive ADMA signal. The lower panel showed loading controls for recombinant UBAP2L and immunoprecipitated PRMT1. **d** Co-IP/WB analysis of the associations of the UBAP2L deletants with PRMT1. HEK293 cells were transfected with Flag-tagged UBAP2L-WT or indicated deletants, followed by treatment with/without AS (500 μM, 1 h), in combination with/without RNase A in the lysis buffer. **e** Co-IP/WB analysis of the associations of the UBAP2L mutants with PRMT1 and ADMA levels. HEK293 cells were transfected with Flag-tagged UBAP2L-WT or indicated mutants (depicted in Supplementary Fig. S1), followed by treatment with/without AS (500 μM, 1 h). The Flag precipitates were analyzed by WB for PRMT1 and ADMA. **f** SG competence in UBAP2L-KD HeLa cells exposed to the same treatments as in (**e**), using Flag (red)/eIF4G (green) as SG markers. **g** Quantification of SGs in (**f**). **h** SG competence in UBAP2L-KD HeLa cells with Flag-tagged UBAP2L-WT or the indicated point mutants (depicted in Supplementary Fig. S1). **i** Quantification of SGs in (**h**). **j** Co-IP/WB analysis of the associations of the UBAP2L point mutants with PRMT1 and ADMA levels in UBAP2L-KD HEK293 cells. *n* = 3. Scale bars = 10 μm

Co-IP/MS analysis also identified another binding partner for UBAP2L, PRMT1 (Fig. 5a). Co-IP/WB analysis further confirmed this interaction (Fig. 5b), supporting the previous finding [31]. In vitro methylation assay showed that UBAP2L is dimethylated by PRMT1 (Fig. 5c). Consistently, PRMT1 knockdown significantly decreased the endogenous UBAP2L ADMA level, and PRMT1 overexpression resulted in an obvious increase in the UBAP2L ADMA signal (Supplementary Fig. S7a). The UBAP2L binding partner G3BP1 is dimethylated by PRMT1 [20]; therefore, to preclude its interference, we repeated the Co-IP experiments after knocking down G3BP1/2. The results showed that G3BP1/2 downregulation negligibly altered the UBAP2L ADMA level (Supplementary Fig. S7a), suggesting that UBAP2L dimethylation by PRMT1 is independent of G3BP1/2. Deletion of the RGG domain, but not others, abolished the UBAP2L interaction with PRMT1 as well as the ADMA signal. Deletion of the UBA domain strikingly strengthened their association (Fig. 5d). These data suggest that the RGG motif in UBAP2L is the unique target region for PRMT1.

To confirm that the arginine residues in the RGG motif are methylated by PRMT1, we designed UBAP2L constructs in which all arginine residues within the RGG motif were mutated to alanine (UBAP2L-R_131–190_A) or lysine (UBAP2L-R_131–190_K) (Supplementary Fig. S1). Alanine substitution was used to test the biological role of arginine residues, whereas lysine substitution was used to test the role of the asymmetric dimethyl functional group, since lysine residues can functionally mimic unmethylated arginine residues but are not recognized by PRMT1 for methylation. The results showed that only the UBAP2L-WT, but not the UBAP2L-R_131–190_A or UBAP2L-R_131–190_K mutants, were targeted and asymmetrically dimethylated by PRMT1 (Fig. 5e). AS treatment decreased the interaction of UBAP2L-WT with PRMT1, as well as the ADMA level (Fig. 5e), indicating that stress decreases UBAP2L methylation. UBAP2L△UBA recruited a greater amount of PRMT1 and had a stronger ADMA signal, whereas in UBAP2L△UBA-R_131–190_K, both PRMT1 binding and the ADMA signal vanished (Supplementary Fig. S7b). This finding further highlighted that the RGG motif in UBAP2L is exclusively targeted by PRMT1.

We then assessed the relationship between arginine methylation and SG formation. SG competence was comparable between UBAP2L-R_131–190_K and UBAP2L-WT, whereas UBAP2L-R_131–190_A displayed a clear SG deficiency (Fig. 5f and g). To specify the arginine residues that are methylated by PRMT1, Arg-187, and Arg-190, two sites with potential ADMA modification [40], were singly or both mutated to Ala (R_187_A, R_190_A, and R_187+190_A, respectively) (Supplementary Fig. S1). However, no significant change in SG formation was observed under normal or AS-stressed condition (Fig. 5h and i), and the PRMT1 association and ADMA signal were still detected (Fig. 5j). Taken together, these data demonstrated that the RGG motif in UBAP2L was asymmetrically dimethylated by PRMT1.

### Decreased arginine methylation in UBAP2L promotes SG assembly

**Fig. 6:**
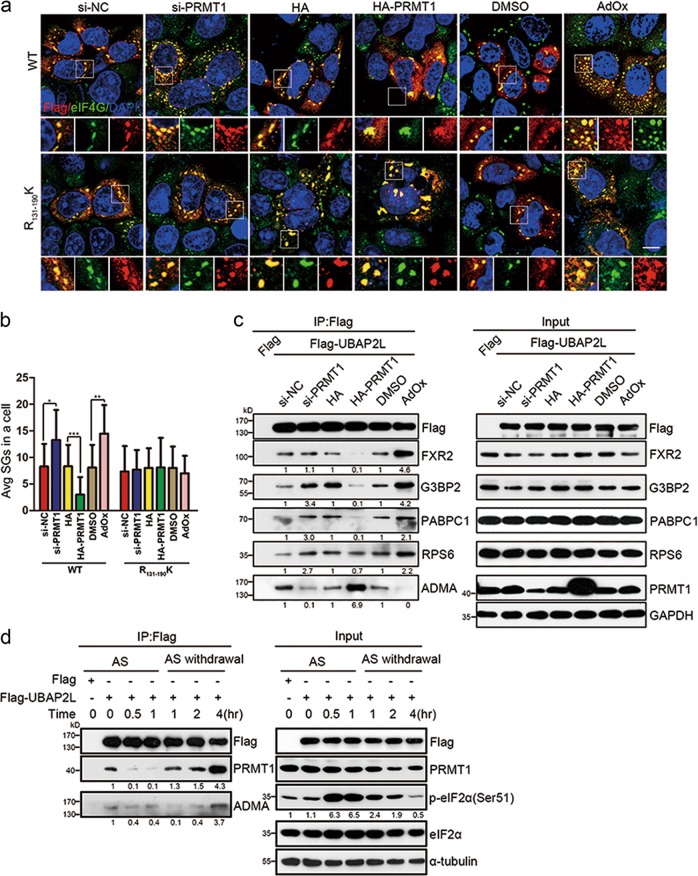
PRMT1 governs arginine methylation of UBAP2L to monitor SG dynamics. **a** SG formation in UBAP2L-KD HeLa cells expressing the Flag-tagged UBAP2L-WT or UBAP2L-R_131–190_K mutants with the indicated treatments. UBAP2L-KD HeLa cells were transfected with *PRMT1*-siRNA or HA-PRMT1 plasmid, or treated with Adox, adenosine dialdehyde, a pan methyltransferase inhibitor (20 μM, 24 h), and then stressed in AS (500 μM, 1 h), using Flag (red)/eIF4G (green) as SG markers. Scale bars = 10 μm. **b** Quantification of SGs in (a). Bars indicate the average number of SGs in Flag-positive cells, and differences between groups were analyzed using the *t*-test. **P < 0.01, *n* = 3. **c** Co-IP/WB analysis of the associations of UBAP2L with SG proteins in HEK293 cells transfected with *PRMT1*-siRNA or HA-PRMT1 plasmid, or treated with Adox. **d** UBAP2L association with PRMT1 and its ADMA level through stress and recovery periods. HEK293 cells expressing Flag or Flag-UBAP2L-WT were stressed with 500 μM AS for 1 h, and then recovered for indicated periods. PRMT1 and ADMA levels were determined by immunoblotting analysis of Flag precipitates. p-eIF2α (S41) in the lysates was shown as a proxy for translation inhibition during the stress and recovery periods

Based on the above results, we hypothesized that decreased arginine methylation in UBAP2L promotes SG assembly. As shown in Fig. 6a and b, PRMT1 knockdown significantly promoted, whereas PRMT1 overexpression significantly suppressed, SG formation in HeLa cells with UBAP2L-WT. However, in the UBAP2L-R_131–190_K group, PRMT1 knockdown or overexpression had no effect on AS-induced SGs. In cells treated with a pan methyltransferase inhibitor, adenosine dialdehyde (Adox), the UBAP2L-WT group, but not the UBAP2L-R_131–190_K group, showed significantly more SGs than the control group (Fig. 6a and b). This suggested that UBAP2L arginine methylation inhibited, whereas its decrease promoted SG assembly. Mechanistically, PRMT1 downregulation increased and PRMT1 overexpression decreased the UBAP2L ADMA level, and increased UBAP2L binding to SG nucleators and RPS6 (Fig. 6c). Correspondingly, Adox treatment eliminated the UBAP2L ADMA signal, and largely enhanced UBAP2L associations with nucleators and RPS6 (Fig. 6c). These observations indicate that arginine methylation inhibited, whereas its decrease facilitated UBAP2L interactions with SG elements. We finally performed a stress-recovery experiment to monitor the methylation status of UBAP2L throughout the treatment and recovery periods. AS stress increased eIF2α phosphorylation, followed by time-dependent degradation after recovery (Fig. 6d). AS treatment decreased PRMT1 association and ADMA levels on UBAP2L, and during recovery, UBAP2L was progressively methylated with ADMA by PRMT1 (Fig. 6d). This suggested that stress decreases UBAP2L methylation, which increased again during stress recovery. Taken together, these results demonstrated that PRMT1 is an important molecular switch that plays a role in the regulation of SG dynamics by monitoring UBAP2L arginine methylation.

## Discussion

In this study, we investigated the cellular roles of UBAP2L in the regulation of SG assembly and disassembly and explored the underlying molecular mechanisms. We summarize our key findings as follows: (1) overexpression of UBAP2L nucleated SGs under the stress-null condition; (2) UBAP2L interacted with SG nucleators and RPs through its RGG motif, which was essential for SG competence; (3) the DUF domain-mediated UBAP2L binding to the G3BP1/2 NTF2-like domain, and its deletion caused UBAP2L shuttling from the cytoplasm to the nucleus, thereby compromising SGs; (4) the RGG motif in UBAP2L was asymmetrically dimethylated by PRMT1; (5) stress decreased UBAP2L methylation, which increased again after stress recovery; and (6) arginine methylation inhibited, whereas its decrease enhanced UBAP2L interactions with SG elements, which subsequently compromised and promoted SG assembly, respectively. These results collectively establish a new role for UBAP2L in RNP biogenesis and RNA metabolism.

**Fig. 7:**
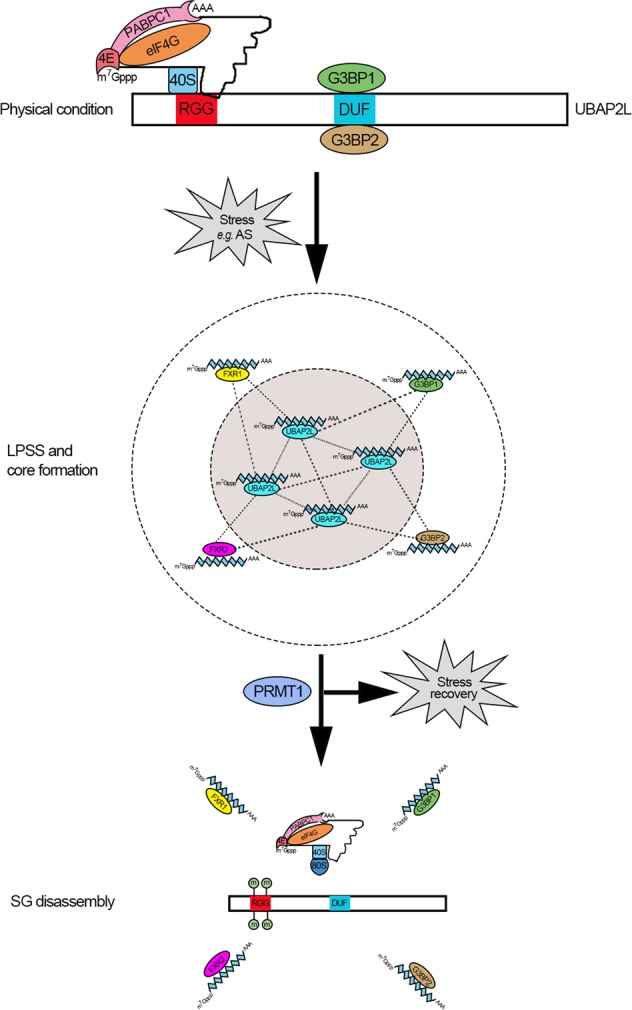
Possible model of the mechanism by which UBAP2L modulates SG assembly and disassembly. UBAP2L recruits mRNPs and other SG-nucleating proteins (G3BP1/2 or FXR1/2) to induce LLPS and initiate core formation for SGs, which are finely monitored by PRMT1

Although the ubiquitin–proteasome system is known to be involved in SG formation [41], our data indicated that the UBA domain, or in other words, the ubiquitin-associated function, was somewhat dispensable for UBAP2L-mediated SG formation, despite the involvement of UBAP2L in ubiquitin signaling (Supplementary Fig. S8). We showed that the RGG motif is required for the effect of UBAP2L on SG competence, as it mediated the interaction of UBAP2L with SG elements, including SG-nucleating proteins and RPs, and hence the nucleation of SGs. The absence of the RGG motif completely abolished SGs. This finding supports the key role of the RGG motif in SG assembly [42]. These features also distinguish UBAP2L from other SG-nucleating proteins, such as TIA-1/TIAR, which have canonical sequence-specific mRNA-binding properties to nucleate SG assembly [43, 44]. The UBAP2L RGG box is enriched in disorder-promoting amino acids [2], including Gly (21 in 60 residues), Arg (19/60), Ser (5/60), Pro (3/60), Glu (3/60), Ala (1/60), Gln (1/60), and Lys (1/60) (Supplementary Fig. S1). This amino acid composition qualifies the RGG motif as an intrinsically disordered protein region (IDPR) [2]. We propose that under normal conditions, the DUF domain mediates strong molecular interactions between UBAP2L and G3BP1/2 by binding to the NTF2-like domain. The RGG motif has weak interactions with mRNPs. Upon stress, the aggregation of stalled mRNPs via the RGG motif in UBAP2L induces LLPS and triggers core formation. UBAP2L also recruits other SG-nucleating proteins (G3BP1/2 or FXR1/2) through conventional protein–protein interactions, which act synergistically to promote the growth and maturation of SGs (Fig. 7).

In this study, deletion of the DUF domain caused UBAP2L shuttling from the cytoplasm to the nucleus. However, a recent study in which the phenylalanine–glycine (FG) repeat was mutated to leucine–alanine (LA) in the DUF domain did not observe such UBAP2L nuclear translocation [32]. It is possible that the FG–LA mutation does not, whereas complete DUF deletion does, impair the UBAP2L intrinsic cytoplasmic localization signal or its interactions with potential cytoplasmic docking proteins. The biological role of UBAP2L shuttling into the nucleus is unknown. We initially thought that there may be a negative mechanism by which UBAP2L recognizes the absence of the DUF domain as reduced expression of G3BP, as UBAP2L regulates G3BP1 transcription [39]. However, double knockdown of G3BP1/2 failed to induce such UBAP2L nuclear translocation (Fig. 4f).

Another important finding of this work is that the methylation status of the RGG motif in UBAP2L was finely monitored by PRMT1 in response to cellular stress. UBAP2L disassociated from PRMT1 concomitant with decreased methylation and in concert with SG assembly under stress, which was followed by re-binding and re-methylation by PRMT1 after stress recovery (Fig. 7). Mechanistically, arginine methylation suppressed, whereas its decrease promoted, UBAP2L association with SG nucleators and RPs, through which PRMT1 modulated SG dynamics. These observations support the previous notion that the RGG motif and arginine methylation are implicated in nucleic acid binding, protein–protein interactions, and signal transduction, except the nuclear/cytoplasmic shuttling [17, 42], which, instead, was directed by the DUF domain. When Arg-187 and Arg-190, two sites in the RGG box with potential ADMA modification, were both mutated to Ala, the UBAP2L ADMA signal and SG formation did not completely vanish. This indicated that other arginine residues in the RGG box may also have the ADMA modification. The possibility also exists that besides ADMA, UBAP2L may harbor SDMA mediated by a different type of methyltransferase, which is likely to be PRMT5, as it methylates G3BP1/2 [20, 21]. Whether an arginine demethylase, such as jumonji domain-containing domain 6 [45, 46], is involved in the regulation of UBAP2L assembly remains to be determined. The physiological or pathological correlation of UBAP2L-involved SGs with related diseases is an important issue. As germ cells are also rich in RNA granules [47–49], we examined the possible correlation between aberrant expression of UBAP2L and spermatogenic defects in patients with azoospermia. The result indicated that reduced UBAP2L expression or assembly in the testis was positively associated with azoospermia (Supplementary Table S3). Further investigation is urgently needed to clarify this issue.

## Supplementary information

Supplementary Data
