# Supplementary material for: UBAP2L arginine methylation by PRMT1 modulates stress granule assembly
Source: Cell Death Differ. 2019 May 21;27(1):227–41. doi: 10.1038/s41418-019-0350-5 (PMC7205891; doi:10.1038/s41418-019-0350-5)
Supplement: Supplementary file 1 — Supplementary Data [file 41418_2019_350_MOESM1_ESM.pdf]

Supplementary Data

Supplementary Methods

8 Supplementary Figures

3 Supplementary Tables

Supplementary Methods

SDS-PAGE and in-gel digestion

Immunoprecipitates were eluted from the beads by incubation with SDS loading buffer in boiling water bath for 10 min and then separated by SDS-PAGE. For in-gel digestion, gels were first visualized using a silver staining kit (Thermo Fisher Scientific, Waltham, MA, USA), and then targeted gel lanes were picked and digested with trypsin.

High-pH reversed-phase chromatography

The Ultimate 3000 HPLC system (Dionex, USA) equipped with a 2.00-mm-inner diameter *100-mm-long Gemini-NX 3u C18110Acolumns (Phenomenex, USA) was used for High-pH fractionation. Peptides were loaded onto the column and washed isocratically at 95% eluent A (20 mM $HCOONH_4$, 2M NaOH) (pH10). The tryptic peptides fractionation was performed using a linear binary gradient from 15% to 50% B (20 mM $HCOONH_4$, 2 M NaOH, 80% ACN) (pH 10) at 0.2ml/min for more than 45 min. Finally, the column was washed at 90% B for 10 min and returned to 95% A for 10 min. Set the UV detector was at 214/280 nm, and fractions were collected every 1 min. 10 fractions were pooled and dried by vacuum centrifuge for subsequent nano-reversed phase liquid chromatography (nano-LC) fractionation.

RPLC-MS/MS analysis

The fraction was resuspended in loading buffer (0.1% FA,2% ACN) and separated with an Ultimate 3000 nano-LC system equipped with a C18 reverse phase column (100-μm inner diameter, 10-cm long, 3-μm resin from MichromBioresources, Auburn, CA). Separate the peptides with the following parameters: 1) mobile phase A:0.1% FA, 5% ACN, dissolved in water; 2) mobile phase B: 0.1% FA, 95% ACN; 3) flow rate: 300nl/min; 4) gradient: B-phase increased from 5% to 40%,70min. Then, the LC eluent was subject to Q Exactive (Thermo Fisher) in an information dependent acquisition mode. MS spectra were acquired across the mass range of 400-1,250 m/z in high resolution mode (> 30, 000) using 250 ms accumulation time per spectrum. A maximum of 20 precursors per cycle were chosen for fragmentation from each MS spectrum with 100 ms minimum accumulation time for each precursor and dynamic

exclusion for 20 s. Tandem mass spectra were recorded in high sensitivity mode (resolution > 15,000) with rolling collision energy on. Raw data were searched against the Uniprot human protein database using the SEQUEST algorithm embedded in the Protein Discoverer 1.3 Software (Thermo Fisher Scientific, Waltham, MA, USA). The following parameters were applied during the database search: 10 ppm precursor mass error tolerance, 1 Da fragment mass error tolerance, static modifications of carbamidomethylation for all cysteine residues, flexible modification of oxidation modifications for methionine residues, and one missed cleavage site of trypsin was allowed. FDR <0.01 was used as filtering criteria for all identified peptides. Only proteins identified with two or more unique peptides were considered, and proteins identified with the same set of peptides were grouped.

Percutaneous testicular sperm aspiration (TESA) and tissue microarray

To perform TESA, the area around the spermatic cord was locally anesthetized by injecting 5 mL of 2% lidocaine. The aspiration was then performed in the center as well as in the upper and lower poles of each testis using a 23-gauge needle with a 20 cc syringe attached to it. A constant negative pressure was applied to the syringe when the needle reached the center of the testis, and aspiration was done with gentle back and forth movements of the needle at different angles in each puncture location. TESA was subsequently performed on both testes. Tunica albugina and epididymis were exposed through a 5- to 15-mm incision in the scrotal skin and tunica vaginalis. Three different incisions were made in tunica albugina near the sites of needle insertion, and a sample of roughly 5×3×2 mm was excised from each site. One specimen from one testis was placed in separate tubes containing IVF medium for intracytoplasmic sperm injection (ICSI). The other one from the other testis was immediately fixed in modified Davidson’s fixative and processed to paraffin embedding. Twenty-nine patients with non-obstructive azoospermia (NOA, with spermatogenic defects; median age 31.5) were included as the experimental group, and twenty-six patients with obstructive azoospermia (OA, normal spermatogenesis; median age 31.5) were as the control group. The specimens were finally processed to a tissue microarray by Shanghai Outdo Biotechnology Co., Ltd (Shanghai, China).

The manipulation was approved by the Ethics Committee of the Third Affiliated Hospital of Guangzhou Medical University, and was conducted according to the principles of the Declaration of Helsinki. Written informed consent was obtained from all participants.

Immunohistochemistry (IHC) and staining evaluation

After deparaffinization and rehydration, tissue microarray slides were heated in a microwave oven for 15 minutes in 10 mM citrate buffer (pH 6.0) and incubated with 3% hydrogen peroxide for 20 minutes, follow by incubation with the primary antibody for UBAP2L (1:200), and the secondary HRP-conjugated goat anti-rabbit IgG (H+L) (Jackson Immunoresearch, West Grove, PA, USA), and then visualized with diaminobenzidine. The slides were finally counterstained with hematoxylin. Each case was evaluated by estimating the percentages and intensity of tumor cells showing a cytoplasm staining pattern. For IHC assessment, the entire tissue section was scanned to assign scores. The staining intensity was scored as 0 (achromatic color), 1 (pallide-flavens), 2 (deep yellow), or 3 (brown). The extent of staining was scored as 0 (<5%), 1 (5%-25%), 2 (26%-50%), 3 (51%-75%), or 4 (>75%), according to the percentages of positively stained areas in relation to the whole tissue area. The sum of the staining intensity and extent scores were used as the final staining scores (0-8). For the purpose of statistical evaluation, tissues with a final staining score of 0 were considered negative (-), 1-2 were weakly positive (+), 3-5 were moderately positive (++) and 6-8 were strongly positive (+++). The difference of UBAP2L expression intensity between the control and NOA group were analyzed by the nonparametric Mann-Whitney singed-rank test (SPSS 13.0).

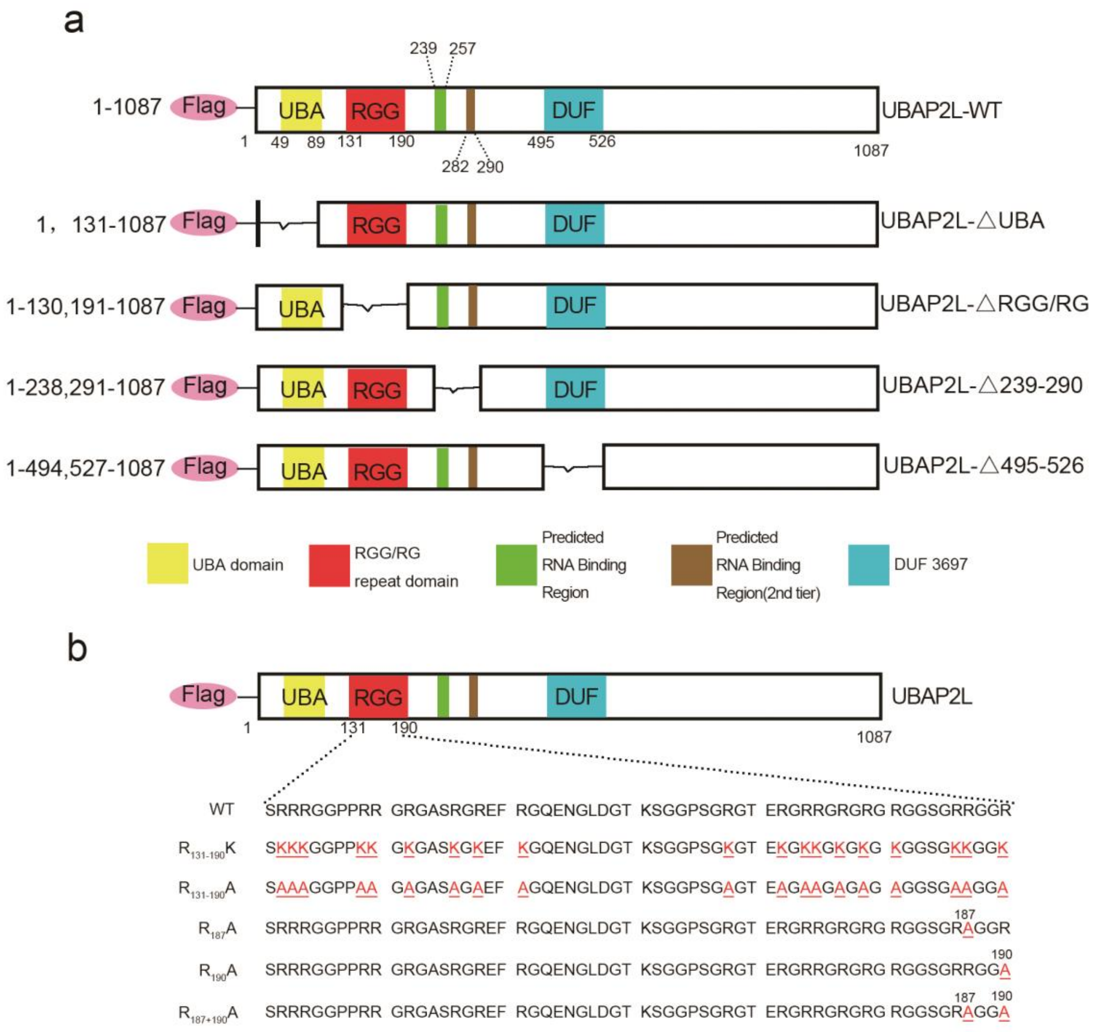

Supplementary Figure S1. Schematic of constructs used in the study. (a) Protein domains in UBAP2L wild-type (WT) and indicated deletants. Amino acid residue numbers appear at the left. (b) Amino acid sequences of the RGG motif in UBAP2L-WT and indicated mutants. Substituted residues are underlined and colored.

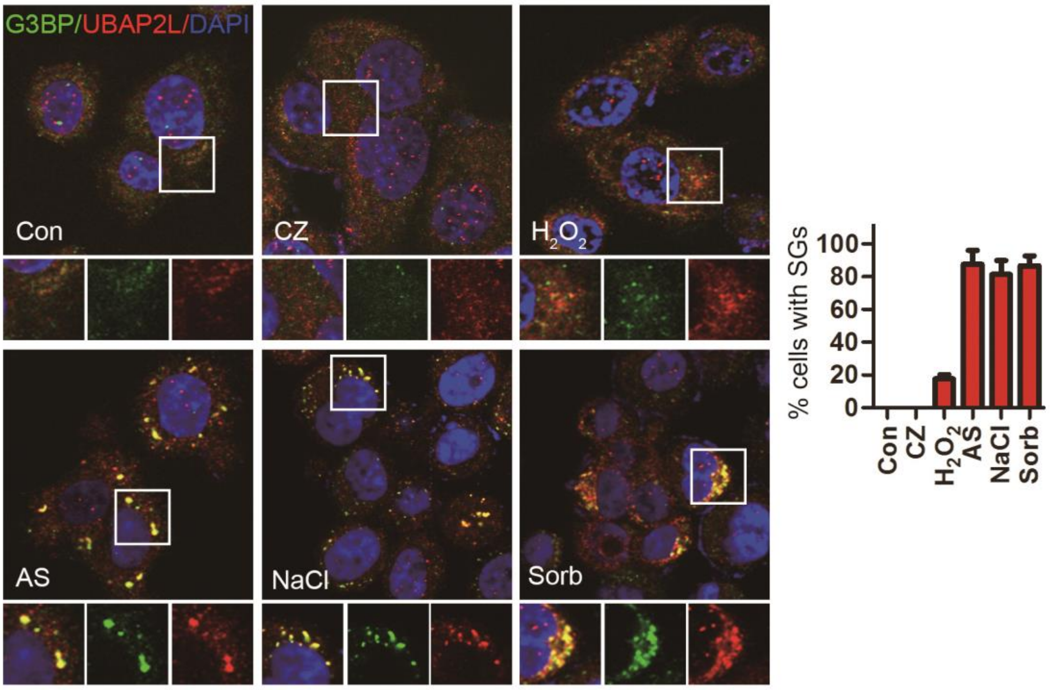

Supplementary Figure S2. Co-immunofluorescence of UBAP2L and G3BP in PC3 cells after indicated treatments. PC3 cells were untreated or stressed with 20 μM CZ, 1 mM H_2O_2, 500 μM AS for 1 h, or 400 mM sorbitol, 200 mM NaCl for 30 min, and then stained for UBAP2L (red)/G3BP (green) as SG markers and scored. Blue indicates nucleus counterstained by DAPI. Inserts are also showed with separated colors. Bars indicate average percentage of cells containing more than three SGs. Data represent the mean ± SD. n = 3. Scale bars = 10 μm.

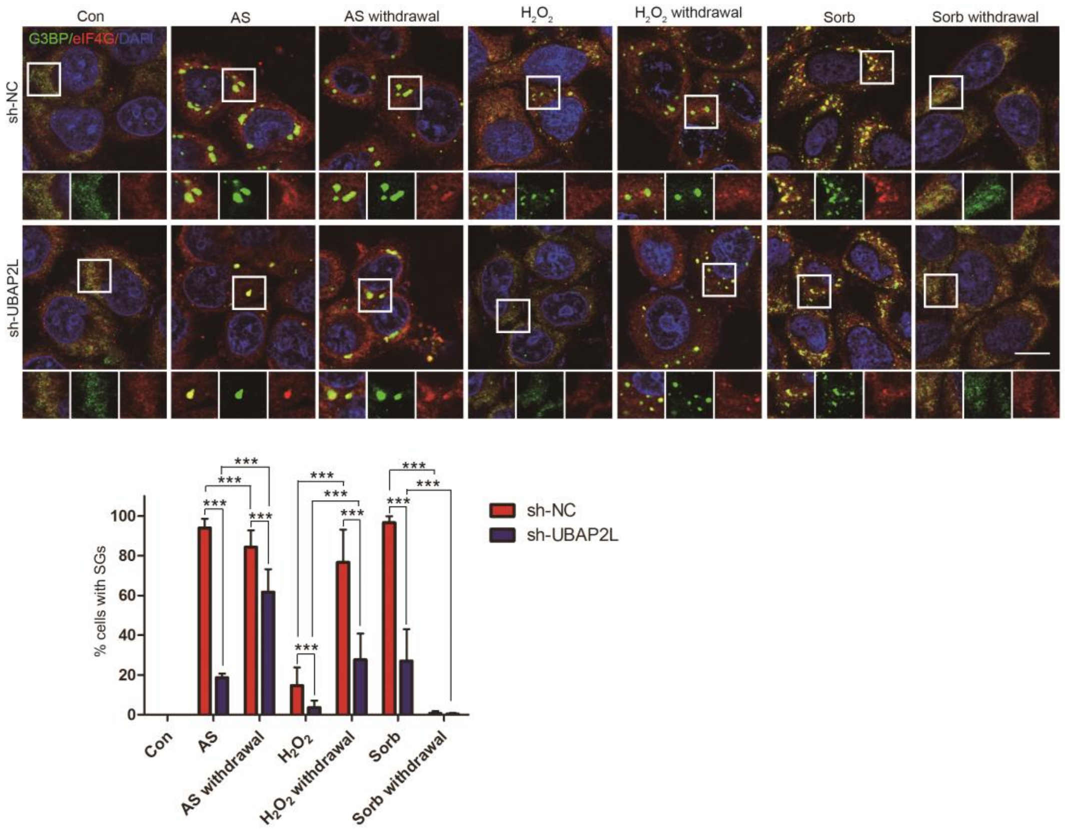

Supplementary Figure S3. SG formation in various stresses or recovery after UBAP2L knockdown by shRNA. HeLa cells were transfected with NC- or *UBAP2L*-shRNA, followed by untreatment or exposure to AS (500 μM, 1 h), H_2O_2 (1 mM, 1 h) or sorbitol (400 mM, 30 min), or recovery from stress for 1 h, and then stained for G3BP (green)/eIF4G (red) as SG markers and scored. Bars indicate average percentage of cells containing more than three SGs. Between-group differences are analyzed using the Chi square test. Data represent the mean ± SD. ***, $P < 0.001$. $n = 3$. Scale bars = 10 μm.

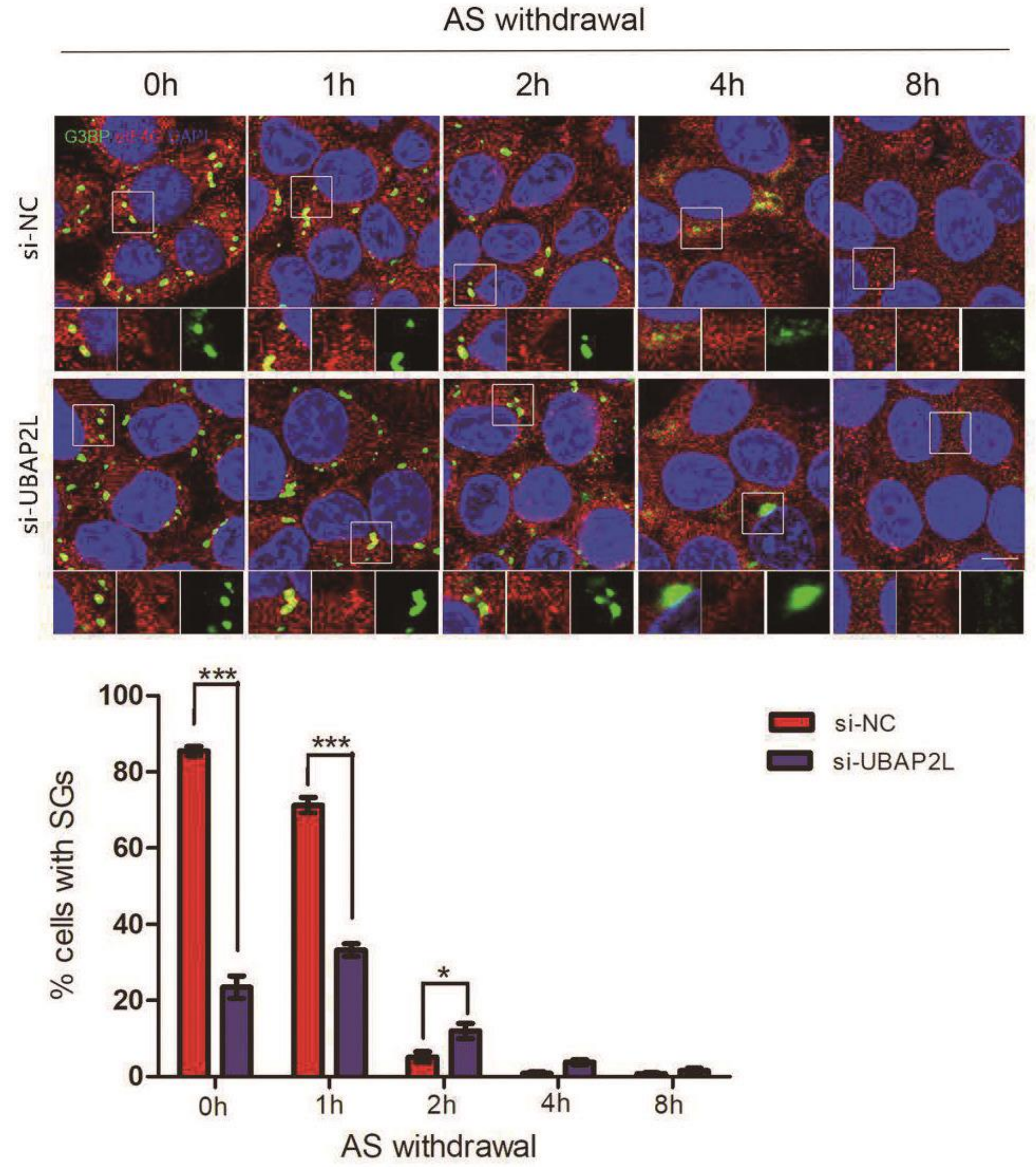

Supplementary Figure S4. Time-course examination of SG disassembly after AS withdrawal in HeLa cells with UBAP2L knockdown. HeLa cells were transfected with NC- or *UBAP2L*-siRNA, followed by treatment with 500 μM AS for 1 h, and then recovery from stress for 1-8 h. SGs were visualized by IF using G3BP (green)/eIF4G (red) as markers and scored. Bars indicate average percentage of cells containing more than three SGs. Between-group differences are analyzed using the Chi square test. Data represent the mean ± SD. *, $P < 0.05$, ***, $P < 0.001$. $n = 3$. Scale bars = 10 μm.

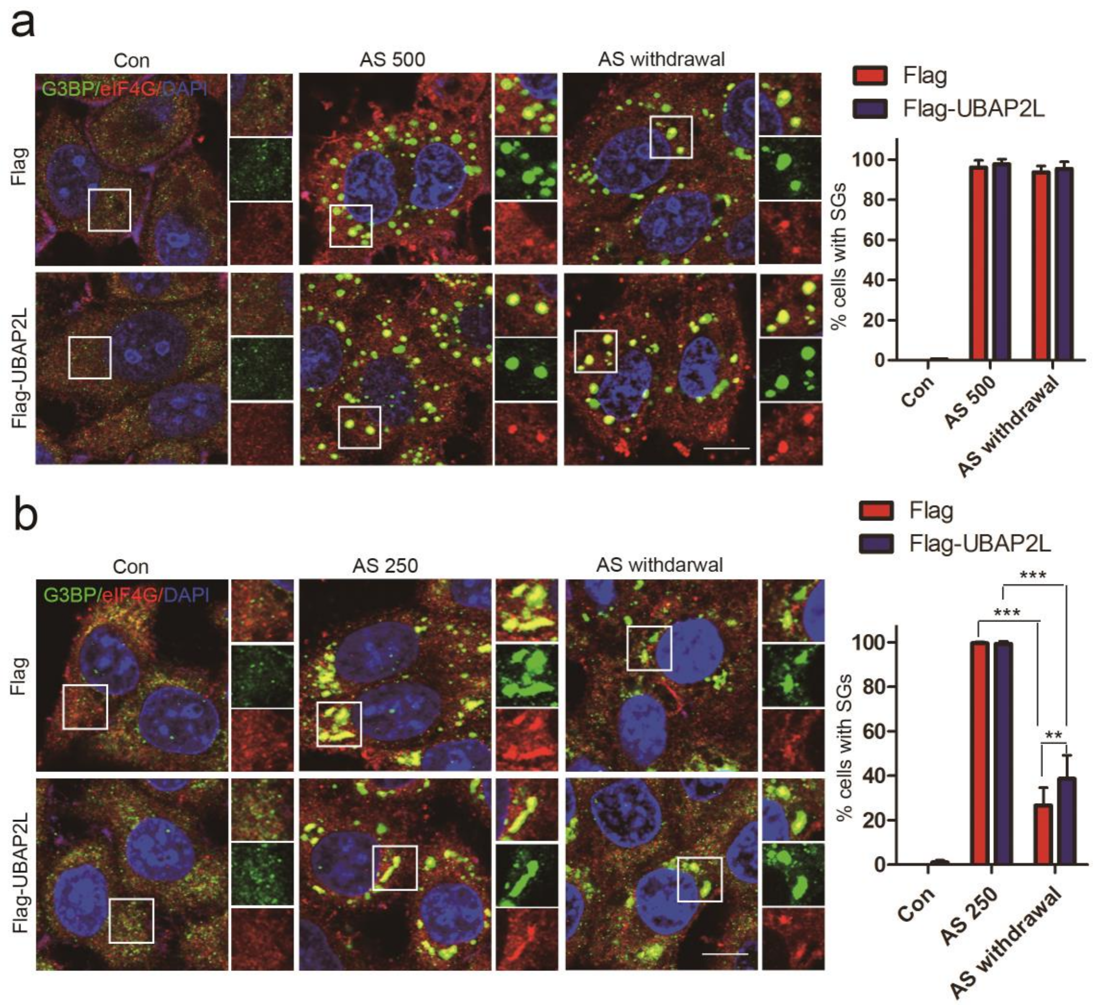

Supplementary Figure S5. SG formation in HeLa cells stressed in different AS concentration or recovery after UBAP2L overexpression. HeLa cells were transfected with Flag tag or Flag-UBAP2L plasmid, followed by treatment with 500 (a) or 250 (b) μM AS for 1 h, or recovery for 1 h, and then stained for G3BP (green)/eIF4G (red) as SG markers and scored. Bars indicate average percentage of cells containing more than three SGs. Between-group differences are analyzed using the Chi square test. Data represent the mean ± SD. **, $P < 0.01$, ***, $P < 0.001$. $n = 3$. Scale bars = 10 μm.

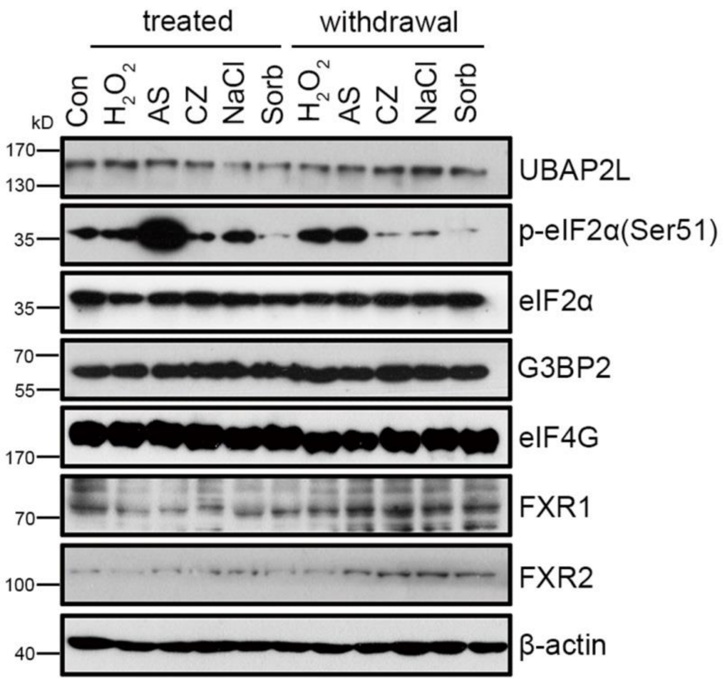

Supplementary Figure S6. UBAP2L expression in PC3 cells after stress or recovery. PC3 cells were treated with or relieved from indicated stresses, and then subjected to WB analysis for UBAP2L, its binding partners and other indicated SG proteins.

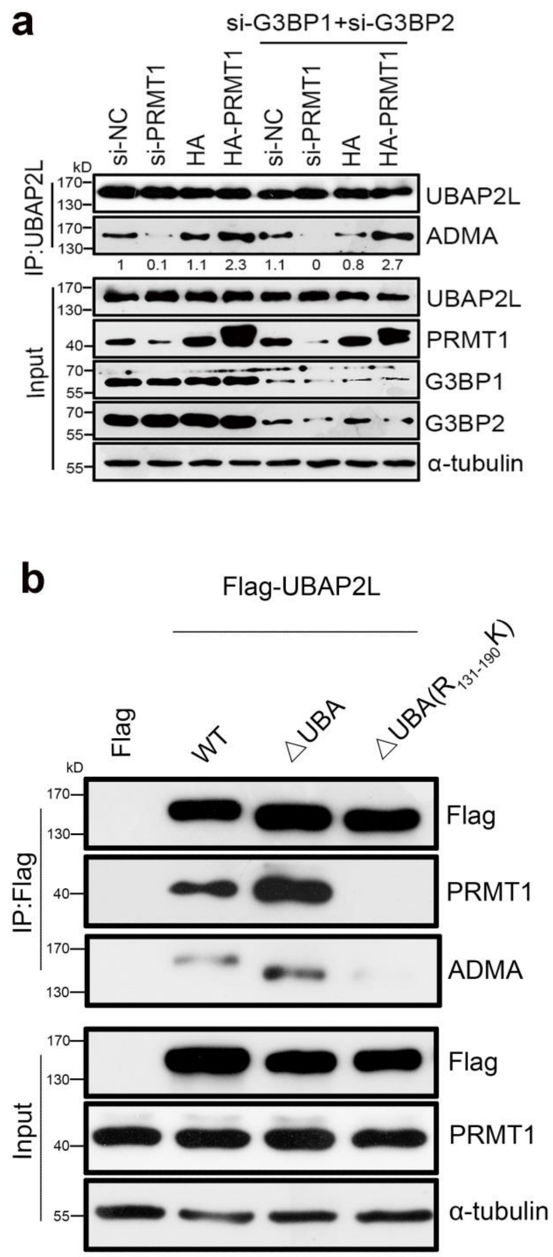

Supplementary Figure S7. (a) UBAP2L ADMA levels after UBAP2L knockdown or overexpression. Endogenous UBAP2L was immunoprecipitated from whole cell lysates of HEK293 cells transfected with *PRMT1*-siRNA or NC- siRNA, HA tag or HA-PRMT1 plasmid, in combination with/without G3BP1/2-siRNA, followed by WB analysis of ADMA modifications on UBAP2L. **(b) Co-IP/WB analysis of UBAP2L-WT or mutants associations with PRMT1 and ADMA levels.** HEK293 cells were transfected with Flag-tagged UBAP2L-WT, UBAP2L∆UBA or UBAP2L∆UBA(R$_{131-190}$K). The Flag-precipitates were analyzed by WB for PRMT1 and ADMA.

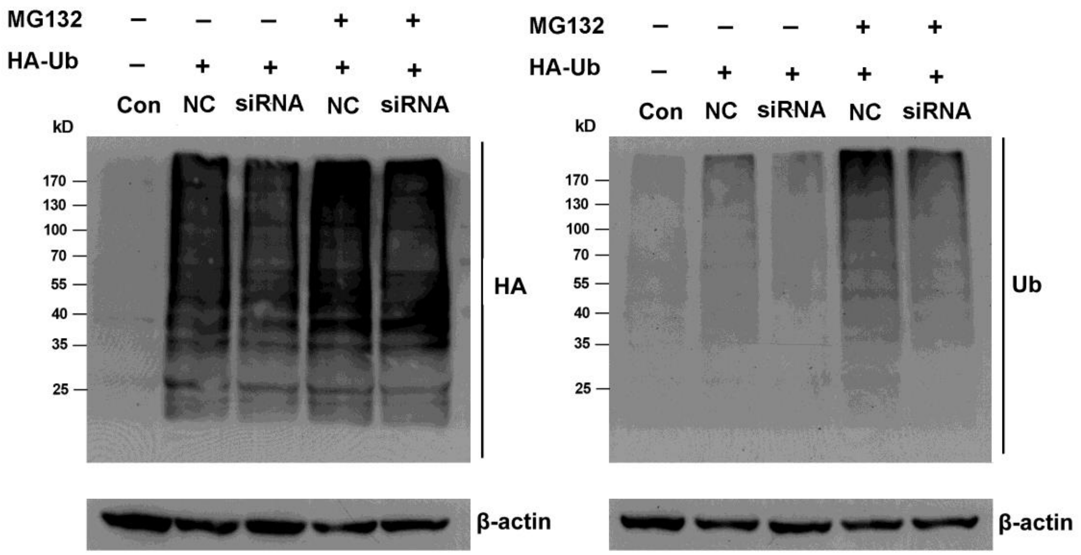

Supplementary Figure S8. Effects of UBAP2L knockdown on intracellular ubiquitin signaling. HeLa cells were transfected with NC- or *UBAP2L*-siRNA, in combination with HA-Ubiquitin (Ub) plasmid, followed by treatment with/without 10 μM MG132 (a proteasome inhibitor) for 10 h. Cell extracts were then analyzed by WB for HA or Ub with a whole gel length.

Supplementary Table S1. siRNA sequences

Gene	Sense (5'→ 3')	Antisense (5'→ 3')
UBAP2L#1	GCCUGUCCUUUCUGAUUAUTT	AUAAUCAGAAAGGACAGGCTT
UBAP2L#2	GCCACAAGUAUAUGGUUAUTT	AUAACCAUAUACUUGUGGCTT
G3BP1#1	CAUUAACAGUGGUGGGAAATT	UUUCCCACCACUGUUAAUGTT
G3BP1#2	ACCUCAUGUUGUUAAAGUATT	UACUUUAACAACAUGAGGUTT
G3BP2#1	UGAAGGAUCUGUUCCAAAUTT	AUUUGGAACAGAUCCUUCATT
G3BP2#2	CAGUGAAUGUCAUACUAAATT	UUUAGUAUGACAUUCACUGTT
PRMT1#1	GUUCCAGUAUCUCUGAUUATT	UAAUCAGAGAUACUGGAACTT
PRMT1#2	GCCUACUUCAACAUCGAGUTT	ACUCGAUGUUGAAGUAGGCTT

Supplemental Table S2. Antibodies.

Antibody	Vendor	Catalog number	Application	Working Dilution for WB or (IF) [IP]
Mouse anti-β-actin	Ray Antibody Biotechnology, Beijing, PRC	RM001V	WB	1:6000
Rabbit anti-ADMA	Cell Signaling Technology, Shanghai, PRC	13522	WB	1:1000
Rabbit anti-eIF2α	ABclonal, Shanghai, PRC	A9905	WB	1:1000
Rabbit anti-p-eIF2a (S51)	ABclonal, Shanghai, PRC	AP0745	WB	1:1000
Goat anti-eIF4G	Novus Biologicals, Minneapolis, MN, USA	AF4018	WB, IF	1:4000 (1:100)
Mouse anti-Flag tag	Sigma-Aldrich, Shanghai, PRC	F1804	WB, IF, IP	1:5000 (1:200) [1:125]
Rabbit anti-FXR1	ABclonal, Shanghai, PRC	A5942	WB	1:2000
Goat anti-FXR1	Abcam, Cambridge, MA, USA	ab51970	IF	(1:100)
Rabbit anti-FXR2	Abcam, Cambridge, MA, USA	ab168852	WB	1:1000
Mouse anti-GAPDH	Ray Antibody Biotechnology, Beijing, PRC	RM2002V	WB	1:6000
Mouse anti-G3BP	BD biosciences, Franklin Lakes, NJ, USA	611126	IF	(1:100)
Rabbit anti-G3BP1	Zen Biosciences, Chengdu, PRC	505342	WB	1:2000
Rabbit anti-G3BP2	Abcam, Cambridge, MA, USA	ab86135	IF	(1:100)
Rabbit anti-G3BP2	ABclonal, Shanghai, PRC	A6026	WB	1:1000
Rabbit anti-HA tag	Sigma-Aldrich, Shanghai, PRC	H6908	WB, IF, IP	1:5000 (1:200) [1:125]
Rabbit anti-Lamin B1	Proteintech, Wuhan, PRC	12987-1-AP	WB	1:2000

Rabbit anti-PABPC1	ABclonal, Shanghai, PRC	A0516	WB	1:500
Rabbit anti-RPL4	Proteintech, Wuhan, PRC	11302-1-AP	WB	1:2000
Rabbit anti-PRMT1	Cell Signaling Technology, Shanghai, PRC	2449	WB	1:1000
Mouse anti-RPS6	Cell Signaling Technology, Shanghai, PRC	2317	WB	1:4000
Mouse anti-α-tubulin	Ray Antibody , Beijing, PRC	RM2007V	WB	1:6000
Rabbit anti-UBAP2L	Bethyl, Montgomery, Alabama, USA	A300-534A	WB, IF, IP	1:2000 (1:100) [1:100]

WB, western blot; IF, immunofluorescence; IP, immunoprecipitation

Supplementary Table S3. Correlation of UBAP2L expression with non-obstructive azoospermia (NOA)

Scale	Control (*n* = 26)	NOA (*n* = 29)	*P* value
Negative (-)	1/26	0/29	0.022
Low (+)	4/26	15/29	
Moderate (++)	15/26	11/29	
High (+++)	6/26	3/29	

A nonparametric Mann-Whitney singed-rank test was used to test the difference between control and NOA.
